# Prophylactic use of recombinant ADAMTS-13 during pregnancy for congenital thrombotic thrombocytopenic purpura

**DOI:** 10.1016/j.rpth.2025.102687

**Published:** 2025-01-21

**Authors:** Éloïse Colliou, Agnès Ribes, Clotilde Gaible, Mathilde Marlas, David Ribes, Isabelle Labadens, Paul Guerby, Stanislas Faguer

**Affiliations:** 1 Department of Nephrology and Organ Transplantation, National Referral Centre for Rare Kidney Diseases, University Hospital of Toulouse, Toulouse, France; 2 Laboratory for Hematology and Hemostasis, University Hospital of Toulouse, Toulouse, France; 3 Department of Clinical Pharmacology, University Hospital of Toulouse, Toulouse, France; 4 Department of Obstetrics, University Hospital of Toulouse, Toulouse, France; 5 Faculty of Health, University Toulouse-3, Toulouse, France; 6 Institute for Metabolic and Cardiovascular Diseases (Unit 1297), National Institute for Health and Medical Research, Toulouse, France

**Keywords:** ADAMTS-13, congenital and thrombotic thrombocytopenic purpura, pregnancy, prophylaxis

## Abstract

**Background:**

Congenital thrombotic thrombocytopenic purpura (cTTP) related to ADAMTS-13 deficiency is associated with a maternal risk of death of 10% and a risk of fetal loss greater than 50% without treatment.

**Key Clinical Question:**

Is prophylactic use of recombinant (r)ADAMTS-13 during pregnancy in patients with cTTP safe and effective in preventing cTTP relapse?

**Clinical Approach:**

rADAMTS-13 was given intravenously weekly (40 Units/kg) from 17 weeks’ gestation. ADAMTS-13 activity was undetectable before the first administration, reached 60% to 90% of normal levels 2 hours after, and became undetectable between days 4 and 6. A full dose was given in the hours preceding the delivery and on day 3. No flare-up of cTTP occurred during the pregnancy, and rADAMTS-13 was tolerated well. No anti–ADAMTS-13 antibodies developed.

**Conclusion:**

Prophylactic use of rADAMTS-13 during pregnancy may prevent relapse of cTTP and reduce the risk of fetal loss, but an optimal regimen requires further attention.

## Essentials

• rADAMTS-13 prevents cTTP relapse, but data on pregnant women are lacking.
• This is the first case of preventive rADAMTS-13 supplementation in a pregnant woman with cTTP.
• rADAMTS-13 infusion was well tolerated and prevented cTTP relapse during pregnancy.
• No immunization against ADAMTS-13 developed during or after pregnancy.

## 1 Introduction

Congenital thrombotic thrombocytopenic purpura (cTTP) is an extremely rare recessively inherited disease linked to the mutation of *ADAMTS-13*, a gene coding for a von Willebrand factor (VWF)-cleaving protease (ADAMTS-13) [1]. ADAMTS-13 deficiency leads to the accumulation of ultralarge VWF multimers that promote platelet-rich microthrombi. Combined with precipitating factors such as pregnancy, ADAMTS-13 deficiency ultimately leads to cTTP, a form of thrombotic microangiopathy (TMA). ADAMTS-13 supplementation with regular fresh frozen plasma (FFP) prevents most cTTP flare-ups [1].

In patients with cTTP, an acute flare-up during pregnancy is associated with a maternal risk of death of 10% and a risk of fetal loss greater than 50% [1]. The probability of live births increases with the gestational age from 37% during the first and second trimesters to 94% during the third trimester [2]. Beyond usual symptoms of TTP, like strokes, the clinical presentation of cTTP during pregnancy (ie, without prophylactic treatment) includes recurrent pregnancy losses, intrauterine growth restriction, and hemolysis/elevated liver enzymes/low platelet count syndrome [[3], [4], [5]].

In patients with recurrent or severe allergy to FFP, plasma-derived factor VIII concentrates, which contain trace amounts of ADAMTS-13, have been utilized [6,7], but the impact on ADAMTS-13 activity is marginal. No other therapeutic alternatives have been approved. Recently, an interim analysis of the *cTTP* phase 3 study reported that recombinant (r)ADAMTS-13 may be an effective prophylactic strategy for patients with cTTP with a good safety profile [7], but pregnant women were excluded from this study. To date, only 2 studies have reported the successful use of rADAMTS-13 as salvage therapy in women with severe flare-ups of cTTP during pregnancy, in combination with FFP infusions [8] or as a single therapy [9]. In this article, we report the first case of successful (off-label) prophylactic use of rADAMTS-13 in patients with cTTP during pregnancy in preventing cTTP relapse.

## 2 Methods

We retrospectively reviewed the clinical charts of a woman who received prophylactic infusions of rADAMTS-13 (TAK755, Takeda) during her second pregnancy. ADAMTS-13 activity was estimated using a fluorescence resonance energy transfer assay (fluorescence resonance energy transfer-VWF73; adapted from Kokame et al. [10]) applied to citrated plasma samples.

According to a pivotal study that reported the use of rADAMTS-13 in patients with cTTP outside pregnancy [7], TAK755 was given weekly at a dose of 40 Units/kg of weight, based on the patient’s weight before pregnancy (3000 Units every week). Pharmacokinetic evaluations were performed after the first administration of TAK755 (at 8 weeks’ gestation).

## 3 Results

A 36-year-old pregnant woman (17 weeks’ gestation) with a history of severe and recurrent headaches was referred for TMA in April 2022 with undetectable ADAMTS-13 activity but no evidence of ADAMTS-13 inhibitor. Despite rapid normalization of TMA parameters while receiving prednisolone and plasma exchanges, a severe intrauterine growth restriction was identified, resulting in fetal death at the 23rd gestational week. The placental pathologic investigation confirmed severe maternal vascular malperfusion. Sequencing of the *ADAMTS-13* gene identified a pathogenic variation at the homozygous state in exon 24 (c.3187C>T; p.Arg1060Trp; American College of Medical Genetics class 5). A final diagnosis of cTTP was retained. Further, FFP infusions were complicated by a severe allergic reaction (angioneurotic edema and urticaria). Since no TTP flare-up had occurred outside pregnancy, and in accordance with the wishes of the patient, FFP prophylaxis was withdrawn with no further TMA flare-ups.

In October 2022, the patient was referred for the management of a new pregnancy (7 weeks’ gestation). She rejected the proposed resumption of FFP prophylaxis. A multidisciplinary discussion of the benefit-to-risk ratio led us to propose off-label prophylactic therapy based on weekly infusions of rADAMTS-13. The patient gave her informed consent to “compassionate use” of TAK755 and subsequent pharmacokinetic follow-up. Aspirin prophylaxis was also prescribed to prevent placental vascular complications.

**Figure 1:**
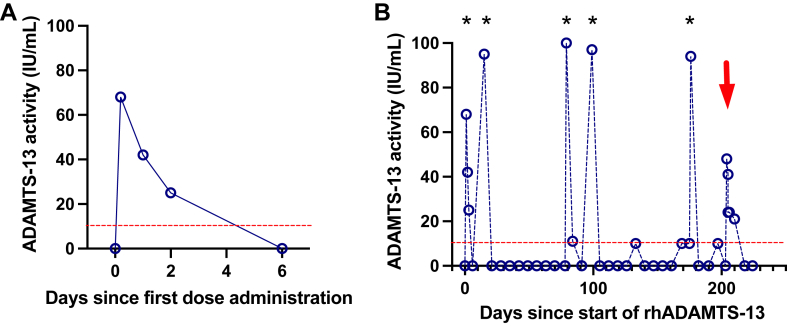
(A) Kinetic profile of ADAMTS-13 activity (percentage of the normal value) after the first administration of TK755 (40 International Units [IU] per kilogram of weight; 3000 IU). (B) ADAMTS-13 activity during the period of ADAMTS-13 supplementation during pregnancy. ∗These values correspond to ADAMTS-13 activity measured 2 hours after the administration of TK755. The red arrow corresponds to the date of delivery. Green arrows correspond to infusions of recombinant ADAMTS-13 before and after delivery. rhADAMTS-13*,* recombinant human ADAMTS-13.

ADAMTS-13 activity was undetectable before the first administration, reached 60% of normal levels 2 hours after, and decreased progressively to become undetectable between days 4 and 6 (Figure 1A). During follow-up, ADAMTS-13 activity measured before the administration of TAK755 (day 7) was undetectable or at the lower limit of quantification for the assay (ie, 10%) but increased up to 90% after administration (Figure 1B).

A full dose (3000 International Units [IU]) was given in the hours preceding the delivery (vaginal route), aiming at an ADAMTS-13 activity greater than 20%. Activity was reassessed daily, and rADAMTS-13 (3000 IU) was reinfused 3 days after the delivery to reduce the risk of a cTTP flare-up after delivery. Thereafter, rADAMTS-13 was infused weekly up to day 30.

Throughout the pregnancy, serum levels of lactate dehydrogenase, troponin-I, and haptoglobin remained within normal ranges, as did platelet values. Regular obstetrical monitoring showed normal fetal growth (46th percentile at week 30) and normal fetal cardiac rhythm. At week 28, the patient had neither hypertension nor proteinuria, but her soluble Fms-like tyrosin kinase-1 (sFLT1) to placental growth factor (PlGF) ratio was slightly elevated (sFLT1/PlGF, 92; normal values < 38). At week 35, she developed proteinuria (urinary protein to creatinine ratio, 1.6 g/g) but had neither hypertension nor increased serum creatinine. Abnormal fetal heart rate but normal growth (25th percentile) were identified. At that time, blood tests showed a normal platelet count and haptoglobin. The sFLT1/PlGF ratio was greatly elevated (ratio, 288), suggestive of a high risk of preeclampsia. A dose of rADAMTS-13 was administered, and delivery was subsequently induced for early preeclampsia. During and after delivery, platelet counts remained within the normal range, and no hemolysis was identified. Pathologic analysis showed an ischemic placenta with distal villous hypoplasia and multifocal infarction lesions. Proteinuria normalized 7 days after delivery.

At birth, the baby was healthy, had no ADAMTS-13 inhibitor, and had a normal platelet count and ADAMTS-13 activity. No sign of hemolysis developed up to discharge from the hospital on day 7.

All infusions of rADAMTS-13 were well tolerated, with no episodes of allergy or fever and no thrombotic or hemorrhagic events. The patient had no headaches. Weekly administration of rADAMTS-13 (3000 IU) was pursued for 1 month after delivery. Maternal breastfeeding was started with no specific complications for the mother or her child. Once rADAMTS-13 supplementation was withdrawn, no flare-ups of TMA occurred. No neutralizing antibodies were detected during or after pregnancy.

## 4 Discussion

In this pregnant woman with cTTP, the first to our knowledge to be treated prophylactically with weekly rADAMTS-13 during her pregnancy, TAK755 was safe and well tolerated and prevented cTTP flare-ups and fetal loss. The efficacy of such a preventive approach in pregnant women with cTTP has yet to be confirmed and adequately assessed in controlled studies. Among the unresolved issues, the optimal dose to be administered remains elusive during pregnancy. Upon administration of rADAMTS-13, the patient had no cTTP flare-ups, but she developed placental vasculopathy with infarction lesions and late features of preeclampsia, another form of TMA. In the absence of information on the pharmacokinetics of TK755 during pregnancy, she was given a standard and constant dose of 3000 IU without reassessment, but pharmacokinetic evaluation suggested incomplete ADAMTS-13 supplementation from days 5 to 7 following TAK755 administration, a finding already observed outside pregnancy (mean time with an increase of ADAMTS-13 activity above 10% of 5.2 days [95% CI, 4.9-5.5]) [7]. Further studies will have to address the optimal regimen for rADAMTS-13 during pregnancy (ie, fixed weight-based doses or readjusted according to predose ADAMTS-13 activity), keeping in mind that standard treatment with FFP delivers a median dose of 8.9 IU/kg compared with 40 IU/kg with rADAMTS-13 [7].

Our patient was at high risk of developing preeclampsia and other placental vascular complications and presented a mild form of late-preterm preeclampsia, resulting in a very positive obstetrical outcome. Although the link between incomplete ADAMTS-13 supplementation and the preeclampsia that developed at week 35 remains elusive, this association was previously described [3,11,12]. In a cohort of 16 women with cTTP and harboring the same mutation of ADAMTS-13, 6 who did not receive prophylactic FFP developed early-onset preeclampsia [3]. Placental examination showed signs of maternal vascular lesions of underperfusion in 13 out of 16 cases. Whether optimization of rADAMTS-13 dosing could have prevented preeclampsia remains uncertain.

In summary, this case strongly indicates the value of compassionate use of prophylactic TAK755 in pregnant women with cTPP when FFP infusions are contraindicated. In the future, rADAMTS-13 may replace FFP infusions in all pregnant women developing cTTP to reduce the risk of anaphylaxis. The optimal regimen of rADAMTS-13 supplementation during pregnancy remains to be defined.
